# Emotional Interdependence and Well-Being in Close Relationships

**DOI:** 10.3389/fpsyg.2016.00283

**Published:** 2016-03-11

**Authors:** Laura Sels, Eva Ceulemans, Kirsten Bulteel, Peter Kuppens

**Affiliations:** Faculty of Psychology and Educational Sciences, KU LeuvenLeuven, Belgium

**Keywords:** emotional interdependence, emotion transmission, well-being, interpersonal relationships, dyadographic

## Abstract

Emotional interdependence—here defined as partners’ emotions being linked to each other across time—is often considered a key characteristic of healthy romantic relationships. But is this actually the case? We conducted an experience-sampling study with 50 couples indicating their feelings 10 times a day for 7 days and modeled emotional interdependence for each couple separately taking a dyadographic approach. The majority of couples (64%) did not demonstrate strong signs of emotional interdependence, and couples that did, showed great inter-dyad differences in their specific patterns. Individuals from emotionally more interdependent couples reported higher individual well-being than individuals from more independent couples in terms of life satisfaction but not depression. Relational well-being was not (relationship satisfaction) or even negatively (empathic concern) related to the degree of emotional interdependence. Especially driving the emotions of the partner (i.e., sender effects) accounted for these associations, opposed to following the emotions of the partner (i.e., receiver effects). Additionally, assessing emotional interdependence for positive and negative emotions separately elucidated that primarily emotional interdependence for positive emotions predicted more self-reported life satisfaction and less empathic concern. These findings highlight the existence of large inter-dyad differences, explore relationships between emotional interdependence and key well-being variables, and demonstrate differential correlates for sending and receiving emotions.

## Introduction

Emotional interdependence, where the feelings of one person are related to the feelings of another person, is often seen as a key characteristic of close relationships. Such interdependence transpires in phenomena like synchrony (concurrent covariation of partners’ emotions; Butler, 2011; Liu et al., 2013; Papp et al., 2013), being moved by one another (coupling; e.g., Boker and Laurenceau, 2007; Butner et al., 2007), emotion transmission (e.g., Larson and Almeida, 1999) and so on. Moreover, such phenomena are often considered defining elements of a healthy romantic relationship. But is this really the case? Is emotional interdependence a characteristic of healthy couples? While past research has certainly provided evidence for the existence of emotional interdependence in romantic couples, the degree and correlates of emotional interdependence remain poorly understood and documented. Yet, identifying the precise nature and correlates of emotion interaction patterns between romantic partners can inspire theory about underlying mechanisms and improve insight in the dynamical interplay of emotions between people. Additionally, it can help to inform research on relational dysfunction, ultimately contributing to the improvement of therapies that focus on emotions, such as emotion focused couples therapy (Johnson et al., 1999). Here, we apply a dyadographic approach to study one aspect of emotional interdependence (one partner’s emotions –the sender- affecting the other partner’s emotions –the receiver- at a subsequent time point), examine its occurrence based on data from real life, and relate this form of emotional interdependence to both individual and relational indicators of well-being, both in general as well as separately in terms of sender- and receiver-effects.

For a long time, emotion research has primarily focused on the intrapersonal aspects of emotions. Emotions mostly originate from interpersonal contexts, however, and research is increasingly shifting attention to how emotions emerge in interactions between people and can become intertwined over time, giving rise to complex interpersonal emotion dynamics (Butler, 2011; Boiger and Mesquita, 2012; Butler and Randall, 2013; Kappas, 2013). These interpersonal emotion dynamics can be observed in several phenomena, one of the most prominent being that one person’s feelings may influence another person’s feelings across time (see Butler, 2011 for an overview). Such a temporally contingent association between people’s emotions has been studied under various names, such as emotion transmission (Larson and Almeida, 1999), emotion contagion (Hatfield et al., 1994; Thompson and Bolger, 1999), susceptibility (Randall and Schoebi, 2015), cross-lagged covariation (Butler, 2011), and crossover (Westman, 2001). Summarizing, we will here refer to it as emotional interdependence.

Emotional interdependence is particularly expected to occur in the context of close relationships, such as parent-infant dyads or adult intimate relationships. It is even often considered one of the defining features of intimate relationships, with partners continuously influencing each other’s emotions, cognition, and behavior (Kelley et al., 1983; Berscheid and Ammazzalorso, 2001; Rusbult and Van Lange, 2003). There indeed seems to be an abundance of evidence for the existence of emotional interdependence in adult romantic relationships, especially for negative emotions (see Larson and Almeida, 1999 for an overview). For instance, stress that one partner experienced at work can crossover to the other partner at home after they get together (Westman, 2001).

Moreover, people seem to consider emotional interdependence as a healthy feature of romantic relationships, emphasizing the necessity of “being on the same wavelength”. Literature about emotional similarity matches this idea, reasoning that tuning emotions to one another has beneficial interpersonal consequences (e.g., Wallbott, 1995; Keltner and Kring, 1998; Anderson et al., 2003, 2004). It would help partners to coordinate their behaviors and thoughts, making them able to collectively respond to situations that demand action. Additionally, it would increase mutual understanding and feeling validated by the partner, promoting social cohesion, attraction, and sympathy. In sum, tuning emotions to one another is expected to be related to relational well-being, and more specifically to relationship satisfaction and empathic concern.

Furthermore, attachment researchers theorize that adult partners “coregulate”, referring to a process in which partners regulate each other’s affect and physiological arousal, resulting in interwoven oscillating emotional patterns (e.g., Field, 1985; Sbarra and Hazan, 2009; Butler and Randall, 2013). By modulating each other’s emotions; partners would help to maintain each other’s emotional stability, which is known to be critical for psychological well-being (e.g., Kuppens et al., 2007; Houben et al., 2015). Hence, based on this literature, we would expect emotional interdependence not only to be related to relational well-being, but also to individual well-being.

Although previous work clearly established the occurrence of emotional interdependence in couples, one may wonder whether it really is a defining feature of optimal close relationships. Indeed, studies explicitly investigating intra- and inter-variability in emotional interdependence suggest that there are substantial inter-individual differences in couples’ interpersonal emotional patterns (Ferrer and Widaman, 2008; Madhyastha et al., 2011; Steele et al., 2014). The specific pattern of emotional interdependence seems to depend on the couple under investigation, with a lot of couples evidencing emotional independence. Currently, several factors have been associated with variation in the amount of emotional interdependence in couples. On a first, macro- level, emotional interdependence is moderated by culture. For instance, Schoebi et al. (2010) found emotional interdependence of anger only in couples that endorse collectivistic values, and not in couples endorsing individualistic values. Additionally, couples in arranged marriages show less emotional interdependence (in the form of synchrony) than couples in love marriages (Randall et al., 2011). On an interpersonal level, the degree of emotional interdependence has been associated with factors such as interpersonal insecurity and perspective taking (Schoebi, 2008), and cooperation (Randall et al., 2013). Also, related literature on covariation of concurrent emotions (called synchrony) suggests that it varies depending on time spent together of the partners (Papp et al., 2013) and relationship quality (Saxbe and Repetti, 2010). On an individual level, emotional interdependence is shown to be moderated by distress (Randall and Schoebi, 2015) and attachment style (Randall and Butler, 2013). Finally, on a micro level, there even seems to be a biological basis for differences in susceptibility for emotional interdependence, as people with a certain variant of the serotonin transporter gene are influenced more by their partner’s affect than others (Schoebi et al., 2012).

Taken together, the aforementioned findings suggest substantial variability in couples’ emotional interdependence. Still, existing research on this topic is limited in a number of important ways. First, emotional interdependence has mostly been investigated in a very confined manner, by looking at connections between the same emotions of both partners (one notable exception is Randall and Schoebi, 2015). Rarely did research take into account that an emotion felt by one partner may affect a different emotion in the other partner, such as for instance emotions of opposite valence. One partner’s negative emotions may increase the other partner’s negative emotions (escalation), but also decrease this partner’s positive emotions (dampening) (Butler, 2011). In other words, a multivariate approach that looks at emotions across different channels is needed to grasp the full dynamic interplay of different emotions.

Second, there is a methodological consideration that existing research on emotion interdependence and its relation with well-being often did not take into account. Until now, research investigating emotional interdependence most frequently adopted a nomothetic perspective (implicit in for instance the actor-partner interdependence model, Kenny et al., 2006), with one model being fitted to data from all couples. However, it is becoming increasingly clear that such a nomothetic approach is no longer tenable because each dyad functions uniquely and nomothetic analyses may fail to map intra-dyad dynamics accurately (Molenaar, 2004; Ferrer and Widaman, 2008; Ferrer et al., 2012, 2013). As a result, Steele et al. (2014) have called for a “dyadographic approach”, in which separate analyses per dyad are taken as starting point and can be used to elucidate possible nomothetic rules. At the same time, a dyadographic approach can only be applied when the emotions of every couple are sampled very intensively; which has become possible only recently. For a long time, studies investigating emotional interdependence in daily life have therefore looked at emotions across days (e.g., Almeida et al., 1999; Thompson and Bolger, 1999; Butner et al., 2007), but in recent experience sampling studies emotions have been assessed four or six times a day for 7 or 10 consecutive days (Schoebi, 2008; Randall and Schoebi, 2015), which not only more closely resembles the tiecourse of how emotions unfold (Butler and Randall, 2013; Butler, 2015), but also allows for a dyadographic approach.

Finally, it remains unclear if emotional interdependence is indeed characteristic of more well-being, both on a relational and individual level. In terms of evidence from emotional similarity and attachment literature, it has indeed been found that emotional similarity during conversations can predict relationship satisfaction in the long term (Anderson et al., 2003; Gonzaga et al., 2007). Yet, there is also research that contests whether emotional interdependence is so desirable. Lab research has repeatedly shown that high interdependence between negative affect, called negative affect reciprocity, can be detrimental for a romantic relationship (Levenson and Gottman, 1983; Gottman, 1998). In daily life, increased linkages of partners’ concurrent negative mood states have also been associated with marital dissatisfaction (Saxbe and Repetti, 2010). However, a recent experience sampling study examining how emotional interdependence is related to well-being in the long term reached different conclusions: Randall and Schoebi (2015) found that individuals whose negative affect was influenced more by their partner’s previous positive affect, showed less distress a year later. The study of Randall and Schoebi (2015) is an important step in gaining more insight into the relationship between emotional interdependence and well-being, as it took into account connections between different emotions, and assessed emotional interdependence on a time scale of multiple times (four) per day. Still, being more or less susceptible for one’s partner (so called receiver effects) are only one angle from which one can investigate the relation between emotional interdependence and well-being. People’s emotions can not only be predicted by the partner’s emotions, but also have the ability to predict the emotions of the partner themselves, reflecting so-called sender effects (Hatfield et al., 1994; Larson and Almeida, 1999). This ability to drive a partner’s emotion could be equally relevant to well-being, because it may increase a person’s sense of efficacy and feeling supported by their partner. Although being a sender has typically been related to individual differences such as gender (e.g., Bolger et al., 1989; Larson and Almeida, 1999), and emotional expressiveness (Hatfield et al., 1994), its relation with well-being remains an untouched area. Based on this reasoning, we want to investigate emotional interdependence and well-being in the couple in general (looking at the combination of receiver and sender effects), as well as to what extent relations with well-being are a function of whether one is one the sending or receiving end of emotional interdependence.

### Present Study

With this study, we aim to obtain a more complete picture of how emotional interdependence takes shape in the daily life of romantic couples and how it relates to well-being. To this end, we conducted an experience sampling study, in which romantic partners simultaneously reported on their emotional experiences 10 times a day for 7 consecutive days. First, to fully grasp emotional interdependence, we applied a multivariate technique to the resulting data of each couple, with both negative and positive emotions of the partner (influence of the partner’s emotions) and the actor (influence of own prior emotions) modeled simultaneously. In this way, we were able to look at cross-partner influences between both similar and different emotions. To prevent the problems associated with a nomothetic approach, we took a dyadographic approach in which we modeled emotional interdependence for each couple separately. Next, we examined how individual and relational well-being indicators relate to being more or less emotionally interdependent; expecting a positive relation between well-being and the degree of interdependence. We focused on two indicators of individual well-being, namely depression and life satisfaction, and two indicators of relational well-being, empathic concern and relationship satisfaction. Additionally, we wondered if it would matter for individuals from a more or less interdependent couple that they are driving or following their partner’s emotions. To assess this question, we made a distinction between sender effects, or the extent to which individuals’ emotions predicted their partner’s emotions over time, and receiver effects, or the extent in which individuals’ emotions were predicted by their partner’s emotions over time, and related these to the well-being indicators. We also assessed if specific characteristics from the couple, such as age and relationship duration were related to the degree of interdependence shown.

## Materials and Methods

### Participants

Couples were recruited through flyers, social media channels, and advertisements in community and relationship therapy centers. Inclusion criteria were: (1) in a relationship for at least two months, (2) heterosexual, (3) over the age of 18, (4) both partners were willing to participate. To obtain a representative sample, we selected couples of varying age, relationship duration, and cohabitation status. In our final sample of 50 couples (100 participants), relationship length varied from 2 months to 35 years (*M* = 72.06 months, *SD* = 107.79 months) and age ranged from 18 to 70 years (*M* = 27.75 years, *SD* = 10.60 years). Ten of these couples were married, 18 couples were not married, but lived together, while 22 couples lived separately. Most participants (94%) had the Belgian nationality and were highly educated, with 60% holding a university degree. Each participant received a monetary compensation of 40 euros for their participation.

### Materials

First, participants completed an online background questionnaire that included demographic questions (e.g., about age and relationship duration) and questions about well-being. Next, participants took part in the experience sampling part of the study, in which they answered questions about their feelings. Descriptive statistics can be found in **Table 1**.

**Table 1:** Descriptive statistics and correlations for key variables.

	Women	Men	Women						Men					
	*M (SD)*	*M (SD)*	NE	PE	D	LS	RS	EC	NE	PE	D	LS	RS	EC
Negative emotions (NE)	10.45 (8.71)	10.12 (8.46)	-						-					
Positive Emotions (PE)	56.66 (10.47)	62.38 (12.07)	-0.44**	-					-0.33*	-				
Depression (D)	1.60 (0.36)	1.50 (0.28)	0.53*	-0.25†	-				0.34*	-0.27†	-			
Life Satisfaction (LS)	5.16 (0.89)	5.53 (0.82)	-0.42**	0.28*	-0.47**	-			-0.23	0.26†	-0.50**	-		
Relationship Satisfaction (RS)	4.24 (0.54)	4.36 (0.47)	-0.50**	0.42**	-0.40**	0.46**	-		-0.15	0.05	-0.22	0.32*	-	
Empathic Concern (EC)	2.89 (0.54)	2.31 (0.62)	0.05	0.12	0.23	-0.21	0.03	-	-0.01	0.07	-0.09	-0.12	0.25†	-

^†^*p <* 0.10*;* *p < 0.05*;* **p < 0.01.

#### Individual Well-Being

Individual well-being was assessed in terms of depression and life satisfaction. To measure depression, the Center for Epidemiological Studies Depression Scale (CES-D; Radloff, 1977) was used. The CES-D consists of 20 items and asks participants to rate how frequently they have experienced depressive symptoms over the past week, on a scale from 0 = *rarely or none of the time [less than one day]* to 3 = *most or all of the time [5–7 days]*. The CES-D is designed for non-clinical samples and has proven to be a reliable and valid measure (Radloff, 1977).

The Satisfaction with Life Scale (SWLS; Diener et al., 1985; Pavot and Diener, 1993) is a 5-item scale measuring participants’ global judgments of own life satisfaction. Participants indicated for each item how much they agreed or disagreed on a 7-point scale, with 1 = *strongly disagree* and 7 = *strongly agree*. This scale shows good convergent validity with other scales and types of assessments of subjective well-being (Pavot and Diener, 1993). In our study, Cronbach’s alphas equaled 0.76 for the SWLS and 0.83 for the CES-D.

#### Relational Well-Being

Relational well-being was assessed in terms of empathic concern and relationship satisfaction. Empathic concern was measured by a subscale of the Davis’ interpersonal reactivity index (Davis, 1983), which consists of seven items that tap “other-oriented” feelings of sympathy and the tendency to experience feelings of warmth, and concern toward others. For instance, one such item asked: “When I see someone being treated unfairly, I sometimes don’t feel very much pity for them.” These items were answered on a 5-point Likert scale ranging from *Does not describe me well* to *Describes me very well*. Empathic concern scores are associated with measures of emotionality and affective empathy (Davis et al., 1994; De Corte et al., 2007). Cronbach’s alpha equaled 0.79.

The Relationship Assessment Scale (RAS; Hendrick, 1988; Hendrick et al., 1998) is a sound, quick measure of global relationship satisfaction about one’s current romantic relationship, consisting of seven items in which respondents answer each item by a 5-point scale ranging from 1 = *low* to 5 = *high*. Example items are “In general, how satisfied are you with your relationship”, “How well does your partner meet your needs?” and “How good is your relationship compared to most?” The RAS has proven to be reliable (Hendrick, 1988; Vaughn and Baier, 1999; Graham et al., 2011), and shows convergent validity through high correlations with relationship satisfaction measures such as the Dyadic Adjustment Scale (Hendrick, 1988; Vaughn and Baier, 1999), the Kansas Marital Satisfaction Scale (Hendrick et al., 1998) and the Couples Satisfaction Index (Funk and Rogge, 2007), and predictive validity by successfully distinguishing between couples that split up and couples that stay together (Hendrick, 1988; Vaughn and Baier, 1999). In this study, Cronbach’s alpha was 0.82.

#### Assessment of Emotions in Daily Life

At each sampling moment, participants indicated how angry, anxious, depressed, sad, relaxed, satisfied, happy, and cheerful they felt by the use of a slider scale (from 0 = *not at all* to 100 = *very much*). These emotions were selected to represent the four quadrants of the affective space, defined by the dimensions of valence and arousal (Russel et al., 1989). We averaged responses to the angry, anxious, depressed, and sad items to create a measure for negative emotion (α = 0.76, within person centered across all time points). Responses to the relaxed, satisfied, happy, and cheerful items were averaged to create a measure for positive emotion (α = 0.83, within person centered across all time points).

#### Contact Between Partners

At each signal, participants were also asked to indicate whether they had had any contact with their partner since the last beep (recoded into 1 = yes; 0 = no). On average, participants individually reported having had contact with their partner in 73% of the assessment moments. We only considered couples as having been in contact if the partners agreed about this, which was the Cases for 88% of the assessment moments (calculated by the number of beeps for which both partners stated they had been in contact or both partners stated they had not been in contact, divided by the number of beeps answered). This resulted in 4317 of the 6465 answered beeps for which couples agreed to have been in contact with their partner (67%).

### Procedure

First, couples received standardized information about the study, gave their informed consent and completed a battery of questionnaires, including the questionnaires described above. Additionally, each partner received a Motorola Defy Plus smartphone programmed with a custom experience sampling application, was familiarized with its use and with how to answer the experience sampling questions.

Subsequently, a signal prompted participants 10 times a day for 7 consecutive days to answer several questions, including the questions about their emotions. The time frame for these signals ranged from 10 AM to 10 PM. The signals followed a stratified random interval scheme with the time frame being divided into 10 equal intervals, and one signal programmed randomly in each interval. On average, signals were separated by each other with 1 h, 12 min, and 12 s (*SD* = 29 min and 2 s). The smartphones were synchronized to ensure simultaneous signaling within each couple, but the order of questions was random within each couple at each beep to avoid cooperation in answering the questions. Compliance was high, with an overall compliance rate of 92.03% (*M* = 64.40 signals, *SD* = 7.15 signals). This study was carried out in accordance with the recommendations of the university’s Social and Societal Ethics Committee.

## Results

### Data-Analytic Procedure

**FIGURE 1:**
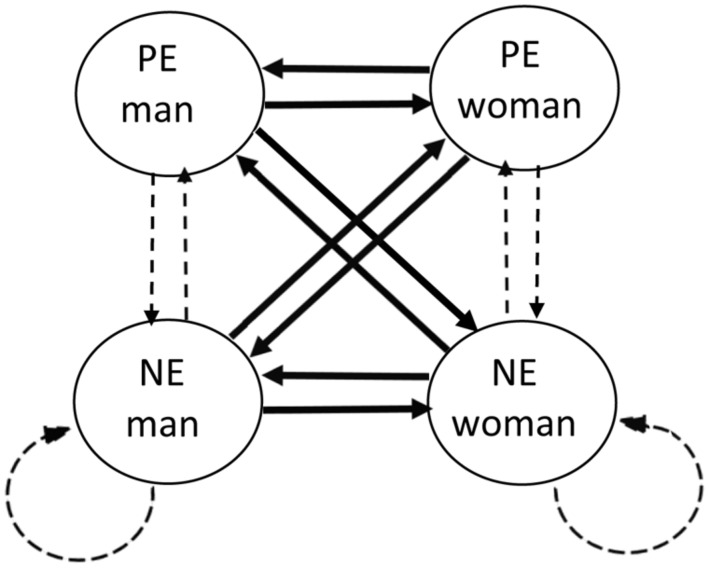
**Interpersonal emotion dynamics presented as a network.** The four nodes represent positive (PE) and negative emotions (NE) of both partners. The arrows represent slopes, and thus the effects of emotions at time *t*-1 on emotions at time *t*. Solid arrows correspond to slopes for cross-partner connections, and thus partner effects, and dashed arrows to within-partner connections or actor effects.

To obtain dyadographic results, we applied lag-one vector autoregressive (VAR) modeling to the data of each dyad separately (Shumway and Stoffer, 2006). VAR models are multivariate versions of autoregressive (AR) models, equipped for modeling time dynamics for multiple variables within a dyad. In these analyses, each variable is regressed on lag-one versions of the same variable and of all other variables. In this way, we can investigate the extent of partner-influence after all intrapersonal effects of each participant are taken into account. Specifically, the self-reported positive or negative emotions of each participant at time *t* is predicted by the positive and negative emotions of both partners at time *t-1*, with time *t-1*, and time *t* referring to two consecutive signals within the same day. For example, the negative emotion of the female partner of couple *i* is regressed on lagged versions of own negative and positive emotion (actor or intrapersonal effects), and her male partner’s negative and positive emotions (partner or interpersonal effects). We first modeled these coefficients and the intercepts as function of two dummy variables, which allowed estimating the effects separately for the moments that couples had been interacting opposed to when they had not. However, five couples did not have enough time points for which they had not been in contact, resulting in missing values for these coefficients. Additionally, in the remaining dyads, there was severe multicollinearity as (1) shown by variance inflation factors above 100 for 35 couples, and above 30 for all couples which is way above the common rule of thumb of ten as cutoff score (Marquaridt, 1970) and (2) the presence of one or more bivariate correlations between predictors above 0.80 in all dyads. Therefore, we decided to not include these dummy variables. As can be seen in **Figure 1**, this approach yields 16 slopes per couple (i.e., 4 times 4), corresponding to the unique direct effects of emotions at time *t*-1 on emotions at time *t*. Eight of these slopes are actor effects, indicating how much one’s emotion is predicted by own emotions at the previous time point (represented by dashed arrows in **Figure 1**). The other eight slopes are partner effects, and model over-time emotional interdependence between partners (represented by solid arrows in **Figure 1**). We performed a sensitivity analysis to evaluate how large the unique effects of the predictors had to be, to be detected by our VAR analysis. Given 48.34 time points (this is the average amount of time points for each couple after taking into account missed signals and signals omitted to avoid over-night relations), the required effect size (f^2^) to reach a power of 0.80 amounts to 0.17. This is considered a medium effect size (Cohen, 1988). We inspected standardized slopes, as they do not reflect the variances of the self-reported emotions (Bulteel et al., 2016).

### Emotional Interdependence

**FIGURE 2:**
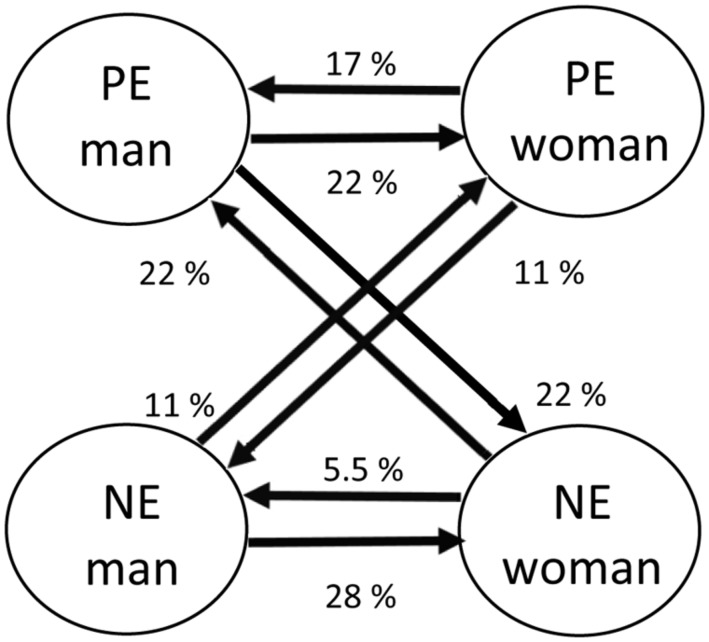
**Percentages of interdependent couples that evidenced specific cross-partner connections**.

To investigate if a couple evidenced substantial interdependence, we used the significance of the slopes representing partner effects as a threshold (with *p* < 0.05). More concretely, if the regression slope for one of the partner effects reached significance, this couple was considered to be substantially interdependent. For the model in which all time points were included, partner effects reached significance in only 18 of the 50 couples (36%). Among these couples, the specific nature of interdependence differed tremendously. For instance, men unidirectionally influenced women in 50% of these couples (*n* = 9), and women unidirectionally influenced men in 39% of the couples (*n* = 7). Bidirectional influence was only evident in 11% of the couples (*n* = 2). Additionally, in some couples, emotions were solely predicted by partners’ similar emotions (50%) while in other couples emotions were solely predicted by partners’ emotions with the opposite valence (28%) and in the remainder, they were predicted by both emotions (22%). Finally, there were some couples in which both negative and positive emotions were predicted by partners’ emotions (11%), and others in which only negative emotions (39%) or positive emotions (50%) were predicted. The direction of the influence could be positive or negative depending on the specific couple, regardless if the emotion was predicted by a similar emotion or by an emotion with the opposite valence. **Figure 2** shows an overview of the percentage of couples in which particular emotional interdependence paths were statistically meaningful. It is noteworthy that women’s positive emotions predicted men’s positive emotions more than that women’s negative emotions predicted men’s negative emotions (17% for positive vs. 5.5% for negative emotions) whereas in men, the opposite pattern was present: men’s negative emotions predicted women’s negative emotions more than that men’s positive emotions predicted women’s positive emotions (22% for positive vs. 28% for negative emotions). We tested if the occurrence of these patterns differed by gender on the basis of Fisher’s exact tests, but these revealed no significant gender differences for sending negative emotions to negative emotions (*p* = 0.10), nor for sending positive emotions to positive emotions (*p* = 0.50).

To investigate the potential influence of shared variance between partner-effects on the magnitude of the unique partner effects, we reran all analyses for VAR-models with three predictors: both the emotions of the actor and only one emotion of the partner. This approach yielded 24 slopes per couple (i.e., three times eight). In the VAR-models with three predictors, partner effects reached significance in 20 of the 50 couples (40%). Hence, shared variance in partner effects could not fully account for the oftentimes weak partner-effects. Additionally, we conducted VAR models in which we only included the time points for which couples stayed in contact with each other in between, to see if this mattered for the amount of interdependence found. On average, this resulted in 31.54 time points remaining for each couple (again, taking into account missed signals and signals omitted to avoid over-night relations). This implies that the required effect size (f^2^) to reach a power of 0.80 now equaled 0.26 which is also considered a medium effect size. Because one dyad only reported to have 15 time points in which they had contact with each other, leading to missing values for their estimated regression coefficients, this couple was excluded from subsequent analyses. For the models in which only the moments were included in which partners remained in contact with each other, again only 18 of the 49 couples showed substantial interdependence (37%), showing tremendous variation in their specific patterns. We therefore decided to focus on the models that took into account all time points, as these models showed higher sensitivity and did not led to the exclusion of couples.

### Emotional Interdependence and Well-Being

To get a sense of the degree of interdependence in each couple, we calculated the density of partner-effects, which stems from the literature on networks (Newman, 2010). As shown in **Figure 1**, over-time emotion dynamics can be represented in a network where each emotion constitutes a separate node and the arrows between the nodes indicate the over-time unique effects of the emotions on one another (Bringmann et al., 2013; Pe et al., 2015). The overall density of the network is a measure of the strength of over-time connections between these nodes. Specifically, this can be calculated by taking the mean absolute strength of temporal connections between all the emotions. As we were only interested in the density of partner-influences, we calculated the average of the absolute values of the eight slopes that represented partner effects for each dyad. This measure makes us able to capture emotional interdependence in a comprehensive way by taking into account interactions between different emotions and in every direction of both partners. The density measure is hence a between-dyad variable, being the same for each partner of the couple but differing across couples.

Next, we wanted to investigate the relationship between well-being indicators and this density measure. To accommodate the dyadic nature of the data, we applied multilevel models in which the couple was the unit of analysis (Kenny et al., 2006). Each well-being indicator was regressed on the couple’s density, and gender was included as a main effect and an interaction effect, in this way allowing and testing for gender differences. As can be seen in **Table 2**, there were, however, no gender differences apparent. Error variance was permitted to differ for men and women. We conducted preliminary analyses in which we added the average amount of contact couples reported to have during the study as a control variable, but it did not affect any of our findings (nor evidenced a main effect on the well-being outcomes), so we dropped it from the final analyses.

**Table 2:** Density of partner-effects and relations with well-being.

						Interactions with gender				
	*B*	*SE*	*T*	*p*	95% CI	*B*	*SE*	*T*	*p*	95% CI
**Individual well-being**										
CES-D										
Intercept	1.72	0.12	14.61	<0.01	1.48;1.95	0.02	0.10	0.18	0.86	-0.18;0.22
Density	-1.22	0.83	-1.48	0.15	-2.90;0.44	0.21	0.71	0.30	0.77	-1.22;1.64
Life satisfaction										
Intercept	4.68	0.29	16.00	<0.01	4.09;5.27	0.14	0.28	0.49	0.63	-0.42;0.69
Density	4.91	2.07	2.38	0.02	0.8;9.06	-2.37	1.96	-1.21	0.23	-6.32;1.58
**Relational well-being**										
Empathic concern										
Intercept	3.16	0.21	15.05	<0.01	2.74;3.58	0.09	0.17	0.52	0.61	-0.26;0.43
Density	-4.12	1.48	-2.78	<0.01	-7.10;-1.15	1.50	1.21	1.24	0.22	-0.93;3.93
Relationship satisfaction										
Intercept	4.54	0.21	21.89	<0.01	4.13;4.96	-0.14	0.13	-1.08	0.29	-0.40;0.12
Density	-1.79	1.47	-1.23	0.23	-4.74;1.51	0.59	0.92	0.64	0.52	-1.26;2.45

#### Individual Well-Being and Density

Being part of a more or less emotionally interdependent couple was not related to individuals’ self-reported depression scores (see **Table 2**). However, individuals from couples that were more emotionally interdependent reported higher life satisfaction than individuals from couples who were more independent.

#### Relational Well-Being and Density

The density of partner effects was negatively related to empathic concern. This means that individuals from couples that were more interdependent reported less empathic concern than individuals from less interdependent couples (with no gender differences in this effect). Being part of an emotionally interdependent couple was not associated with individuals’ self-reported relationship satisfaction.

Given that the relation between emotional interdependence and well-being may vary as a function of the type of emotion experienced, we examined relations between well-being and emotional interdependence separately for positive and negative emotions. In first instance, we calculated separate density scores corresponding to cross-partner effects on an emotion with a specific valence. Specifically, the density score for positive emotions was calculated by averaging the four absolute slopes representing cross-partner effects in the VAR models with positive emotions as a dependent variable. The density score for negative emotions was calculated by averaging the four absolute slopes representing cross-partner effects in the VAR models with negative emotions as a dependent variable. Then, again multilevel models were applied. The density for positive emotions was positively related to life satisfaction [β = 4.44, *t* = 3.23, *p* = 0.002, 95% CI(1.68,7.20)] and negatively to empathic concern [β = -2.63, *t* = -2.52, *p* = 0.02, 95% CI(-4.72,-0.53)] but not to depression [β = -0.87, *t* = -1.51, *p* = 0.14, 95% CI(-2.03,0.29)] or relationship satisfaction [β = -0.45, *t* = -0.44, *p* = 0.66, 95% CI(-2.52,1.62)], whereas the density for negative emotions did not relate to any of the well-being variables (all *p* > 0.05). Hence, emotional interdependence only predicted more life satisfaction and less empathic concern when it were the positive emotions that were dependent on the partners’ emotions. To shed more light on the precise processes behind the relation between emotional interdependence and well-being, we next calculated a stricter density for positive emotions by aggregating the absolute slopes of the two cross-partner effects between positive emotions, representing how much partners’ positive emotions were influenced by each other’s positive emotions. The density for negative emotions was calculated in a similar way, by aggregating the absolute slopes of the two cross-partner effects between negative emotions. This revealed that the density for positive emotions was positively related to life satisfaction [β = 2.39, *t* = 2.23, *p* = 0.03, 95% CI(0.23,4.54)], and marginally negative to empathic concern [β = -1.57, *t* = -1.99, *p* = 0.05, 95% CI(-3.16,0.02)], whereas for the density for negative emotions, no relations with well-being outcomes were found.

Additionally, we assessed if emotional interdependence varied with specific aspects of the couple, such as age or relationship duration (relationship duration was averaged across male and female partner’s report, as *r* = 0.99), but found no association with any of these variables [age: β = 19.18, *t* = 0.54, *p* = 0.59, 95% CI (-52.71,91.07)]; relationship duration: *r* = 0.19 *p* = 0.18). We also investigated if couples who lived together showed more emotional interdependence than couples who still lived separately, but found no significant difference in the magnitude of the density scores, with *M*_cohabiting_ _couples_ = 0.14, *SD*_cohabiting_ _couples_ = 0.05, *M*_non-cohabiting_ _couples_ = 0.13, *SD*_non-cohabiting_ _couples_ = 0.04, *t*(48) = 0.21, *p* = 0.83.

### Sending and Receiving, and their Relations with Well-Being

We next examined relations with well-being distinguishing between sender effects that reflected the extent to which an individuals’ emotions predicted their partner’s emotions over time, and receiver effects that reflected the extent to which individuals’ emotions were predicted by their partner’s emotions over time. To this end, we computed two new density measures: The density for sending was calculated by taking the mean absolute values of the four slopes that represented how much each individual’s negative and positive affect predicted their partner’s subsequent negative and positive affect. The density for receiving was calculated by taking the mean absolute values of the four slopes that represented how much each individual’s negative and positive affect was predicted by their partner’s previous negative and positive affect (note that the receiver effects of one partner equal the sender effects of the other partner, and vice versa).

We applied multilevel models in which each well-being indicator was regressed on the receiving and the sending density, including gender as a main effect and an interaction effect with these densities (resulting in so called actor-partner interdependence models; Kenny et al., 2006). Error variance was allowed to differ for men and women. Results can be found in **Table 3**, and as can be seen here, again no gender differences appeared in any of the effects.

**Table 3:** Density sending vs. receiving and relations with well-being.

						Interactions with gender				
	*B*	*SE*	*T*	*p*	95% CI	*B*	*SE*	*T*	*p*	95% CI
**Individual well-being**										
CES-D										
Intercept	1.72	0.12	14.27	<0.01	1.48;1.96	0.03	0.10	0.28	0.78	-0.18; 0.24
Density sending	-0.81	0.55	-1.47	0.15	-1.91;0.29	-0.01	0.55	-0.01	0.99	-1.09;1.09
Density receiving	-0.44	0.54	-0.81	0.42	-1.50;0.63	0.17	0.53	0.32	0.75	-0.89; 1.23
Life satisfaction										
Intercept	4.69	0.30	15.61	<0.01	4.08;5.29	0.03	0.27	0.11	0.92	-0.52;0.58
Density sending	4.46	1.40	3.19	<0.01	1.68;7.24	-1.07	1.39	-0.77	0.45	-3.84;1.70
Density receiving	0.42	1.37	0.31	0.76	-2.31;3.15	-0.82	1.37	-0.60	0.55	-3.54;1.89
**Relational well-being**										
Empathic concern										
Intercept	3.21	0.21	15.24	<0.01	2.79;3.63	0.12	0.17	0.69	0.49	-0.23;0.47
Density sending	-2.77	0.93	-2.97	<0.01	-4.62;-0.91	-0.34	0.92	-0.36	0.72	-2.17;1.50
Density receiving	-1.60	0.94	-1.70	0.09	-3.48;0.28	1.70	0.94	1.82	0.07	-0.16;3.56
Relationship satisfaction										
Intercept	4.59	0.21	21.83	<0.01	4.16;5.01	-0.13	0.13	-0.98	0.33	-0.40;0.14
Density sending	-1.17	0.87	-1.35	0.18	-2.90;0.56	-0.54	0.85	-0.63	0.53	-2.23;1.16
Density receiving	-0.82	0.85	-0.96	0.34	-2.51;0.88	1.09	0.84	1.30	0.20	-0.57;2.75

#### Individual Well-Being and Density

Nor the degree of receiving nor the degree of sending was related to self-reported depression. The density for receiving was not related to life satisfaction, but the density for sending was positively related to life satisfaction. Hence, predicting one’s partner’s emotions more was associated with more life satisfaction compared to predicting one’s partner’s emotions less.

#### Relational Well-Being and Density

The density for receiving was not significantly related to an individual’s empathic concern. Sending density related negatively to empathic concern, thus individuals that influenced their partners more reported less empathic concern. Both the density for sending and the density for receiving were unrelated to individuals’ relationship satisfaction.

#### Validation Analysis

Finally, we performed a validation analysis to establish that the calculated density scores indeed can be considered to represent emotional interdependence between partners over time. We hypothesized that if the density score measures emotional interdependence, breaking emotional interdependence would decrease the magnitude of the density score. To test this, we randomly mixed up the time points of the male member in every couple while keeping the time points of the female partner in the original chronological order, and calculated new VAR models. Hence, our couples remained the same, but density now described cross-partner effects for emotions on different and random time points, on the basis of which no emotional interdependence would reasonably be expected. We expected that this mixing would mainly impact the density scores in emotionally interdependent couples, and not so much in independent couples, since there was not much interdependence in these couples to begin with. Indeed, for the 18 couples that were considered emotionally interdependent by the original analyses, their new density scores were significantly lower than their original scores [*t*(17) = -5.04, *p* < 0.001, *M*_new_ = 0.10, *SD*_new_ = 0.04]. For the independent couples, the magnitude of the new density scores did not differ significantly [*t*(31) = 0.57, *p* = 0.57, *M*_new_ = 0.13, *SD*_new_ = 0.11]. Additionally, both density scores were unrelated to each other across couples (*r* = 0.22, *p* = 0.13), and the new density score did not predict any of our well-being indicators (all *p* > 0.05).

## Discussion

With this study, we aimed to contribute to the understanding of emotional interdependence in couples’ daily life and its relation with well-being. A dyadographic approach revealed that in fact the majority of couples under study did not show strong evidence of emotional interdependence (64%). Contact between partners during the day did not account for this finding because analyses in which only these time points were included, revealed similar results. This finding might seem surprising, but in fact echoes other dyadographic research on variability in emotional interdependence (Ferrer and Widaman, 2008; Madhyastha et al., 2011; Steele et al., 2014). Further, we found that when couples evidenced substantial interdependence, the specific interdependence patterns varied tremendously with associations between every emotion pair and in every direction. Unidirectional interdependence, in which only one partner influences the other partner across time, was the predominant pattern, and only a few couples evidenced bidirectional interdependence. Although it is recognized that emotional interdependence can be unidirectional (e.g., Larson and Almeida, 1999; Ferrer and Nesselroade, 2003; Gottman, 2013), bidirectional interdependence and reciprocity are often considered to be the norm (e.g., Gottman, 2013).

Despite this observed heterogeneity, the strength of emotional interdependence related to one aspect of individual well-being, life satisfaction, and one aspect of relational well-being, empathic concern. Individuals that were part of emotionally more interdependent couples reported higher life satisfaction and less empathic concern than individuals that were part of more independent couples. Assessing emotional interdependence for positive and negative emotions separately elucidated that primarily emotional interdependence for positive emotions predicted more self-reported life satisfaction and less empathic concern. Additionally, analyses in which a subdivision was made between the degree in which an individual’s emotions drove (sender effects) or followed (receiver effects) the partner’s emotions over time, revealed that it consistently was the magnitude of sender effects that was positively associated with life satisfaction, and negatively with empathic concern. For life satisfaction, these findings were consistent with our expectations, as we proposed that driving one’s partner’s emotion would increase individuals’ sense of self-efficacy and feeling supported by their partner, which are known to enhance well-being (Bandura, 1997; Kuijer and De Ridder, 2003; Maisel and Gable, 2009). Meanwhile, the findings for empathic concern were unexpected. Less empathic concern was related to more emotional interdependence in couples, and primarily on each other’s positive emotions, and not to emotional influence on each other’s negative emotion. Based on past research, empathy is expected to be positively related to emotional interdependence, and especially in negative emotions (Hatfield et al., 1994). To explain these results, we think it is important to keep in mind that empathic concern was only related to shaping the partner’s emotions, and not to being susceptible for the partner’s emotions. Although in the past being emotionally influenced by people’s emotions has been related to empathy (more specifically cognitive empathy or perspective taking) (Hatfield et al., 1994; Schoebi, 2008), Hatfield et al. (1994) did argue that driving people’s emotions would be related to different characteristics. More concretely, powerful senders would be people who (1) experience strong emotions, (2) possess the ability to express emotions, and (3)-most relevant here- are insensitive to emotions of others who are feeling different emotions. It also appears that emotions tend to flow from the person that has the most power to the person with less power (Larson and Almeida, 1999; Anderson et al., 2003), and the need for power is inversely related to empathic concern (Koestner et al., 1990). Additionally, although these findings were contrary to our expectations, they converge with another recent counterintuitive finding in which an interpersonal factor associated with less interpersonal concern, namely attachment avoidance (Joireman et al., 2002), was associated with more emotional interdependence in couples (Randall and Butler, 2013).

It is notable that being more or less sensitive for the emotions of the partner, or so-called partner susceptibility (e.g., Randall and Schoebi, 2015), did not relate to well-being in our sample. In the past, existing research often focused on this aspect of emotional interdependence (e.g., Levenson and Gottman, 1983; Gottman et al., 1998; Schoebi, 2008; Schoebi et al., 2010, 2012; Randall et al., 2013; Randall and Schoebi, 2015) or on emotional interdependence for the couple as a whole (by the form of synchrony; Saxbe and Repetti, 2010; Papp et al., 2013), while sender effects largely remained untouched. This type of research has shown both positive (Randall and Schoebi, 2015) and negative relations (Levenson and Gottman, 1983; Gottman et al., 1998; Saxbe and Repetti, 2010) between emotional interdependence and well-being. However, these researchers all investigated emotional interdependence for specific emotion combinations between partners, such as between hard negative emotions (Levenson and Gottman, 1983; Saxbe and Repetti, 2010) or between soft negative emotions and positive emotions (Randall and Schoebi, 2015). In our study, such fine grained distinctions were not assessed. It is possible that being able to influence your partner’s emotions (this is, being a sender) is beneficial in general, whereas the benefits of being susceptible for partner’s emotions depend more on the specific circumstances or ways in which this occurs. Of course, this remains speculative, and needs further investigation.

Our findings have a number of implications for the study of interpersonal emotion dynamics. First, the results about the prevalence of emotionally independent couples and the heterogeneity in emotional interdependence patterns support the importance of applying a dyadographic approach (Ferrer and Widaman, 2008; Madhyastha et al., 2011; Steele et al., 2014). Additionally, they highlight an important direction for future research; examining under which precise circumstances interpersonal emotion connections occur, what forms they take in what couples, and how and when presumed interpersonal regulation processes play a role (Niven et al., 2009; Zaki and Williams, 2013). Further, our study showed that emotional interdependence can be investigated from different angles; by looking at a couple as a whole or by taking an individual perspective, and here by investigating the extent to which an individual is susceptible for the partner’s emotions or the extent in which he/she drives the partner’s emotion. Depending on the angle, we showed differential relations with well-being indicators. This implies the additional value of investigating all three, and of identifying the specific mechanisms that underlie these.

The results of our study should, however, be considered in light of several limitations. For one, we assessed emotions in daily life in which no distinction could be made between emotions whose precipating sources were within the relationship versus emotions whose origins lied outside the relationship. However, emotions directed at the partner can lead to a totally different emotional interaction with the partner than emotions directed toward something external to the relationship (Berscheid and Ammazzalorso, 2001; Parkinson and Simons, 2012; Butler, 2015). Additionally, our findings are correlational and consequently do not speak to the underlying mechanisms of emotional interdependence. It is possible that the change in the receiver’s emotion, following the sender’s emotion, has nothing to do with the previous emotion of the sender (see Larson, 2002). Also, our findings should be interpreted in the context of the timescale on which emotions were measured. Emotional interdependence can be investigated at different timescales, ranging from second-to-second to longer term, and the underlying mechanisms and processes of these dynamics may not necessarily be the same. The use of time lags also implies the necessity of choosing a time interval for these lags. The time span we selected, allows for the occurrence of various non-partner related events that can override any immediate emotional influence of the partner. It should be noted, however, that several studies that find nomothetic evidence for emotional interdependence have used similar or even larger time intervals, such as daily reports (e.g., Thompson and Bolger, 1999; Butner et al., 2007) or four time points per day (Saxbe and Repetti, 2010; Randall and Schoebi, 2015). Additionally, we assessed empathy in our sample by measuring individuals’ empathic concern in general, and not individuals’ specific empathic concern for their relationship partner (this is dyadic empathy). General and dyadic empathy are, however, related to each other (Péloquin and Lafontaine, 2010), and are both associated with relationship satisfaction (Franzoi et al., 1985; Davis and Oathout, 1987; Long and Andrews, 1990), and positive relationship behaviors such as providing social support (Devoldre et al., 2010) and forgiveness to one’s partner (Paleari et al., 2005; Burnette et al., 2009). Still, as noted by Péloquin and Lafontaine (2010), they do not overlap completely, and it is important to see if similar results would be found for dyadic empathy. Finally, our sample was relatively small and homogeneous, in that it consisted mostly of young, highly educated, Western European, unmarried adults, which limits generalizability. For instance, Schoebi et al. (2010) have found that emotional interdependence depends on the endorsement of collectivistic values; with interdependent couples, such as couples from collectivistic cultures, showing more emotional interdependence than couples from more individualistic cultures. This finding may help to explain why we found so little emotional interdependence in our sample; and suggests the need for future research to examine if emotional independence is as prevalent in a more diverse, representative sample of couples. Additionally, one would expect relationship duration to be a moderator of emotional interdependence, with people that are together for a longer time showing more interdependence, although this did not seem to be the case in our sample.

## Conclusion

Examining emotional interdependence and its correlates in a dyadographic way, we drew attention to several important factors of interpersonal emotion dynamics such as the heterogeneity in emotional interdependence, the potential differential relation between interdependence and individual versus relational wellbeing, and differences in sender versus receiver effects. In doing so, the current study forms an important step to an in depth understanding of interpersonal emotion dynamics and how it relates to well-being.

## Author Contributions

LS, EC, KB, and PK all contributed by – Substantial contributions to the conception or design of the work; or the acquisition, analysis, or interpretation of data for the work; AND – Drafting the work or revising it critically for important intellectual content; AND – Final approval of the version to be published; AND – Agreement to be accountable for all aspects of the work in ensuring that questions related to the accuracy or integrity of any part of the work are appropriately investigated and resolved.

## Conflict of Interest Statement

The authors declare that the research was conducted in the absence of any commercial or financial relationships that could be construed as a potential conflict of interest.
